# Autonomous AI systems in the face of liability, regulations and costs

**DOI:** 10.1038/s41746-023-00929-1

**Published:** 2023-10-06

**Authors:** Agustina D. Saenz, Zach Harned, Oishi Banerjee, Michael D. Abràmoff, Pranav Rajpurkar

**Affiliations:** 1 Department of Biomedical Informatics, Harvard Medical School, Boston, MA USA; 2 Department of Medicine, Brigham and Women’s Hospital, Boston, MA USA; 3 Department of Medicine, Harvard Medical School, Boston, MA USA; 4 Center for Artificial Intelligence in Medicine & Imaging, Stanford University, Palo Alto, CA USA; 5 Fenwick & West LLP, Mountain View, CA USA; 6 Department of Ophthalmology and Visual Sciences, University of Iowa, Iowa City, IA USA; 7 Department of Biomedical Engineering, University of Iowa, Iowa City, IA USA; 8 Department of Electrical and Computer Engineering, University of Iowa, Iowa City, IA USA; 9 Digital Diagnostics Inc, Coralville, IA USA

**Keywords:** Health policy, Medical ethics, Machine learning

## Abstract

Autonomous AI systems in medicine promise improved outcomes but raise concerns about liability, regulation, and costs. With the advent of large-language models, which can understand and generate medical text, the urgency for addressing these concerns increases as they create opportunities for more sophisticated autonomous AI systems. This perspective explores the liability implications for physicians, hospitals, and creators of AI technology, as well as the evolving regulatory landscape and payment models. Physicians may be favored in malpractice cases if they follow rigorously validated AI recommendations. However, AI developers may face liability for failing to adhere to industry-standard best practices during development and implementation. The evolving regulatory landscape, led by the FDA, seeks to ensure transparency, evaluation, and real-world monitoring of AI systems, while payment models such as MPFS, NTAP, and commercial payers adapt to accommodate them. The widespread adoption of autonomous AI systems can potentially streamline workflows and allow doctors to concentrate on the human aspects of healthcare.

## Main

In recent years, the proliferation of AI systems in medicine, including FDA clearances for clinical use as AI/ML and Software as Medical Devices, has led to increased adoption^1^. Many of these systems serve as assistive tools for clinicians, but autonomous AI systems can operate independently, completing tasks without human intervention. For example, while a non-autonomous AI tool might assist radiologists by showing the probability that an X-ray shows an abnormality, an autonomous AI system might independently identify normal X-rays and generate reports for them, bypassing radiologists. Autonomous AI systems also fundamentally differ from autonomous systems without AI, such as insulin pumps due to their advanced capabilities. While traditional autonomous systems use relatively simple predefined rules to produce outputs, autonomous AI systems can harness complex models learned through data to make more sophisticated decisions and are governed by more intricate regulatory frameworks.

Key regulatory clearances, such as the FDA’s approval of LumineticsCore for diagnosing diabetic retinopathy and the European Union’s approval of ChestLink for auto-reporting normal chest X-rays, set an important precedent for the future of autonomous AI systems across medical specialties. Recent advances in AI, affecting areas such as generative AI and large-language models, have increased model performance across diverse use cases and will likely further accelerate the development of autonomous AI systems. Although autonomous AI systems have the potential to improve patient and population outcomes, they raise crucial questions around liability, regulations, and costs.

As AI models advance, liability implications become increasingly complex, with sophisticated systems capable of handling complex medical tasks and blurring the line between human and AI decision-making. While some autonomous AI systems have shown performance comparable to or exceeding human experts, mistakes leading to patient harm will inevitably occur. Fully autonomous AI system results or recommendations can still be used or reviewed by healthcare personnel, potentially introducing liability for licensed clinicians, such as an internal medicine physician interpreting a chest X-ray. To establish medical malpractice, it must be shown that the physician breached their duty of care to the patient, typically determined by the physician’s failure to comply with medical custom or act reasonably given the state of medical knowledge at the time. However, physicians may be uncertain about how using autonomous AI systems might affect their malpractice liability exposure.

Based on the material, malpractice risks identified by legal scholars and the empirical literature, we expect that judges and juries should favor physicians in scenarios where they followed the rigorously validated autonomous AI system’s recommendation. Legal scholars have classified eight scenarios involving a physician’s use of an autonomous AI system that are relevant to malpractice liability, and they hypothesized that there are only two scenarios in which the physician might face liability: (A) the system correctly recommends management that corresponds with the current standard of care, and the physician disregards this recommendation, resulting in patient harm; and (B) the system erroneously recommends management that is nonstandard care, and the physician follows this recommendation which results in patient harm^2^. However, the physician’s malpractice risk for using such AI systems is likely mitigated by the following considerations. Firstly, the use of certain autonomous AI systems has actually been deemed standard of care^3^, and we expect to see more such AI systems being granted this status as time goes on. Secondly, simulated “jurors” often reached a different verdict with regard to scenario B, concluding that the physician should actually not be held liable in this situation. Thirdly, in any of the six scenarios contemplated outside of scenarios (A) and (B), judges are likely to decide such suits in the physician’s favor before the matter even gets to the (potentially unpredictable) jury box^4^. So if only scenario (A) presents a material danger to the physician regarding medical malpractice, then trusting in the AI system’s management recommendation should attenuate that risk. However, meaningful clarity on these questions of liability will only come as lawsuits are adjudicated, and precedent is established.

Medical malpractice lawsuits involve negligence on the part of the physician, but liability might also fall on the shoulders of the creator of the AI system, which could be sued for negligently designing or implementing the AI system in a manner that results in patient harm. For example, if the AI creator failed to rigorously validate the AI system according to industry best practices, such as segregating the test dataset from the AI system’s training data, then the AI creator could be sued and held liable for negligence that resulted in patient harm. In addition, AI creators might be liable for breach of contract if the agreement they enter into with hospitals or physicians states that the AI system will perform according to the agreed upon specifications (e.g., the specifications cleared by the FDA), but the AI system fails to do so. AI creators may attempt to mitigate their risk of liability by purchasing insurance to protect against such eventualities and negotiating contractual safeguards, such as limiting their total liability to the hospital or being indemnified when the harm is the fault of the hospital or its personnel.

Another consideration for hospitals and physicians intending to deploy autonomous AI systems is the evolving regulatory landscape, as the FDA is currently making strides to accommodate medical AI systems, including autonomous systems. In January 2021, the FDA released the Artificial Intelligence and Machine Learning Software as a Medical Device action plan, which is intended to support and provide oversight for continued innovation. According to the plan, the FDA will focus on ensuring device transparency, developing methods to evaluate and improve AI systems, running real-world performance monitoring pilots, and further refining its own regulatory framework. In addition, in October of 2021, FDA provided further detail to this action plan in the form of guidance on good machine learning practices (GMLP). They identified ten key principles, including dataset curation, model design, and development to the model’s deployment and monitoring. More recently, the FDA released its draft guidance on the AI/ML Predetermined Change Control Plan.

In addition to the rapid changes witnessed at the government level, significant policy developments are unfolding within non-profit organizations and healthcare systems^5–7^. These collective efforts are dedicated to formulating guidelines and establishing AI governance committees, addressing critical concerns related to transparency, equity, bias, safety, patient privacy, model robustness, and accountability. These committees oversee AI systems throughout their entire lifecycle. They actively evaluate model development, conduct thorough assessments of training datasets to ensure data diversity, oversee shadow deployments for ongoing performance monitoring and fairness evaluation, and provide user training and effective patient communication. For example, they develop resources such as fact sheets for clinicians that provide comprehensive information on the intended use of the AI system, potential risks and warnings, and details about the algorithm’s performance^8^. These resources empower healthcare professionals and patients to make informed decisions and understand the limitations and benefits of AI technologies. Ultimately these committees remain engaged through continuous monitoring to identify performance drops promptly, enabling timely intervention, updates, or modifications to maintain the system’s accuracy, reliability, and safety. These steps are a sign of progress, but further guidance is needed to help ensure that autonomous AI systems are reliable and ethically developed.

Lastly, we need to account for payment models before deploying autonomous models into clinical settings. Currently, there are multiple potential ways to defray costs, including through insurance reimbursement. Once cleared or approved by the FDA, new technologies such as autonomous AI systems that have been shown to improve clinical outcomes have achieved sustained, long-term reimbursement through the Center for Medicare and Medicaid Services (CMS)’s Medicare Physician Fee Schedule (MPFS). As an example, under PFS, in 2020, CMS allows national coverage and reimbursement for the first use of autonomous AI, for the diagnosis of a retinal complication of diabetes through CPT code 92229, and commercial payers have followed CMS’s lead by also covering the service^9^. Alternatively, they may qualify for New Technology Add-On Payments (NTAP) based on a diagnosis-related group (DRG). NTAP makes it possible to obtain temporary reimbursement for new technology that would not otherwise fall under the DRG, since those payments lag behind true costs by 2 or 3 years^10^. And in September 2020, CMS approved reimbursement through NTAP for an AI-driven software that automatically triages patients based on large vessel occlusions seen on CT scans.

Looking beyond reimbursement, there may be other financial incentives to adopt specific autonomous AI systems. If certain systems prove to be the next standard of care, providers who do not use them may face monetary penalties. For example, when digitizing health records became the standard of care, hospitals who did not switch in a timely fashion to electronic health records were penalized by insurers. In addition, autonomous AI systems can defray costs by improving workflow efficiency and improving patient outcomes and health equity^11^. For example, by incorporating autonomous systems for specialized disease diagnosis and treatment, primary care providers in outpatient centers and hospitalists in inpatient centers, these healthcare professionals could effectively decrease the frequency of referrals and consultations^12^. This reduction would result in an improved care model that aligns with the reimbursement framework of value-based care. By reducing reliance on referrals and consultations, healthcare providers can better allocate resources, time, and expertize to patients needing specialized care, enhancing the overall care model. While we are still in the early days of integrating AI, it is promising that regulatory agencies and insurers are quickly adapting to support AI technology. Payment models will continue to evolve as new technologies disrupt the current standard of care.

As autonomous AI systems become increasingly available for various medical tasks, their potential to reduce waste and improve patient outcomes and health equity is becoming evident^13^. For widespread acceptance among healthcare practices and providers, there must be a concerted effort to ensure the ethical and safe development of autonomous AI models. In addition, we must establish equitable patient benefits and robust monitoring protocols tailored to these models’ unique capabilities. In the long run, we anticipate a broad adoption of autonomous AI systems, playing a significant role in streamlining workflows, handling language-related tasks, and freeing doctors to focus more intently on the human aspects of healthcare.
